# A machine-learning guided method for predicting add-on and switch in secondary data sources: A case study on anti-seizure medications in Danish registries

**DOI:** 10.3389/fphar.2022.954393

**Published:** 2022-11-10

**Authors:** Peter Suhr Breitenstein, Israa Mahmoud, Fahed Al-Azzawi, Saeed Shakibfar, Maurizio Sessa

**Affiliations:** Department of Drug Design and Pharmacology, University of Copenhagen, Copenhagen, Denmark

**Keywords:** pharmacoepidemiology, switches, add-ons, machine learning, real-world data

## Abstract

**Purpose:** There is a lack of available evidence regarding the treatment pattern of switches and add-ons for individuals aged 65 years or older with epilepsy during the first years from the time they received their first anti-seizure medication because of the lack of valid methods. Therefore, this study aimed to develop an algorithm for identifying switches and add-ons using secondary data sources for anti-seizure medication users.

**Methods:** Danish nationwide databases were used as data sources. Residents in Denmark between 1996 and 2018 who were diagnosed with epilepsy and redeemed their first prescription for anti-seizure medication after epilepsy diagnosis were followed up for 730 days until the end of the follow-up period, death, or emigration to assess switches and add-ons occurred during the follow-up period. The study outcomes were the overall accuracy of the classification of switch or add-on of the newly developed algorithm.

**Results:** In total, 15870 individuals were included in the study population with a median age of 72.9 years, of whom 52.0% were male and 48.0% were female. A total of 988 of the 15879 patients from the study population were present during the 730-day follow-up period, and 988 individuals (6.2%) underwent a total of 1485 medication events with co-exposure to two or more anti-seizure medications. The newly developed algorithmic method correctly identified 9 out of 10 add-ons (overall accuracy 92%) and 9 out of 10 switches (overall accuracy 88%).

**Conclusion:** The majority of switches and add-ons occurred early during the first 2 years of disease and according to clinical recommendations. The newly developed algorithm correctly identified 9 out of 10 switches/add-ons.

## 1 Introduction

In 2016, about 46 million individuals worldwide were diagnosed with epilepsy (Beghi, 2020; Sen et al., 2020). The age distribution of epilepsy has a peak incidence and prevalence in those aged 65 years or older, affecting both sexes evenly. According to prior research, 70% of older individuals with epilepsy may live seizure-free if they get the appropriate diagnosis and treatment (Devinsky et al., 2016a). Despite the considerable effort over the past decade in health-care practice and research in epilepsy, its pharmacotherapy is far from optimal, especially among older individuals in polypharmacy for which the total risk of death is still three times higher than that observed in the general population (Devinsky et al., 2016a). Current treatment recommendations for individuals aged 65 years or older suggest starting immediately with anti-seizure medication (ASM) monotherapy in the majority of patients. However, only 47% of patients reach a seizure-free status with the first ASM trial (St Louis et al., 2009). If a patient experiences breakthrough seizures while taking moderate dosages of their first ASM trial, increasing the dose of that ASM is acceptable until toxicity or therapeutic inefficacy occurs. If the patient continues to have breakthrough seizures, therapeutic substitution/switch to a second monotherapy or even a third monotherapy is recommended. However, patients who do not achieve seizure freedom after receiving an adequately chosen and ideally given first ASM monotherapy are unlikely to acquire seizure freedom in subsequent ASM monotherapy trials. In fact, only 13% achieve seizure-free status with the second monotherapy trial and even less with a third monotherapy trial (St Louis et al., 2009). When patients fail monotherapy, it is suggested that they have therapeutic add-ons, or pursue non-pharmacologic therapies (St Louis et al., 2009). Despite clear recommendations from clinical guidelines on how to proceed pharmacologically in individuals with new-onset epilepsy, currently, there is a lack of available evidence from real-world data regarding the pharmacological treatments that older individuals with epilepsy had during the first years of the disease. Evidence is even scarcer regarding which extent recommendations from clinical guidelines are followed in this subgroup. This is mostly driven by flaws in available pharmacoepidemiological tools to assess switches and add-ons in secondary data as highlighted in a recently conducted systematic review (Meaidi et al., 2021).

Therefore, this study aimed at filling this gap in knowledge by using Danish administrative and health-care data to investigate the treatment pattern that individuals over 65 years diagnosed with epilepsy had during the first 2 years from the time they received their first ASM. To achieve this aim, a new machine-learning guided algorithm able to identify switches and add-ons in Danish registries was developed.

## 2 Methods

### 2.1 Data sources

The data sources used in the study were the Danish nationwide administrative and health-care registers/databases. A linkage between the different Danish registers is possible since every Danish citizen has a personal identification number. The personal number can be used to retrieve data from the registers information on sex and age (Danish Civil Registration System Register) (Schmidt et al., 2014), medication redemptions in pharmacies (Danish Prescription Register) (Gaist et al., 1997), deaths (Danish Cause of Death Register) (Helweg-Larsen, 2011), and hospital admission/ambulatory visit (Danish National Patients Register) (Lynge et al., 2011). Since 1977, the Danish Health Data Authority has maintained the Danish National Patient Registries, which are updated weekly. This register records diagnoses and procedures performed at Danish hospitals. The variables of interest in the registry are the date of diagnosis and the diagnosis, dates of admission, and discharge. Epilepsy diagnosis was determined *via* this registry (Lynge et al., 2011). Since 1985, Statistics Denmark has been managing the Danish Civil Registration System, which is updated quarterly. In addition, this register includes the Danish Population Register and the Danish Emigration Register. From this register, we retrieved information on the sex, date of birth, emigration date, and personal identification number of the individuals included in the study population (Pedersen, 2011). We used the Danish National Prescription Register to obtain information about redeemed prescriptions for patients with epilepsy in community pharmacies. Since 1970, the Danish Health Data Authority has administered and updated the Danish Cause of Death Register. It contains information on all deaths in Denmark, as well as the cause of death for Danes. The register’s variables of interest are the date of death and the individual’s personal identification number. The date of death was used to censor patients at death (Helweg-Larsen, 2011).

### 2.2 Study population

The study population included inhabitants of Denmark between 01/01/1996 and 01/01/2018 who were diagnosed with epilepsy and redeemed their first prescription for ASMs after epilepsy diagnosis (i.e., incident new-user design). The date of redemption of the first prescription for ASMs served as the temporal anchor for following the study population in Danish registries and was defined as the index date. Individuals included in the study population should have never redeemed ASM before the index date. This study design helps to minimize biases associated with a mix of frequent and incident users of medicines by limiting the study to individuals under observation who begin pharmacological therapy for the first time (wash-out phase) (Danaei et al., 2012). Individuals were categorized as having epilepsy if they had been hospitalized or were receiving outpatient treatment for epilepsy (International Classification of Disease code, ICD-8 code 345, ICD-10 codes G40). In Danish registers, the positive predictive value of epilepsy diagnosis is 81 percent (95% confidence interval, CI: 75–87%) among patients admitted to the hospital (Christensen et al., 2007).

### 2.3 Follow-up period

Individuals included in the study population were followed up for 730 days from the index date or rather the date when they redeemed their first ASM following epilepsy diagnosis until the end of the follow-up period, death, or emigration. A total of 730 days were chosen because previous studies highlighted that most switches and/or add-ons occur within the first 2 years in older individuals aged 65 or older who were treated with ASM for epilepsy (St Louis et al., 2009).

### 2.4 Outcomes

The study outcomes are the overall accuracy of the classification of switch or add-on of the newly developed algorithm.

### 2.5 Algorithm

The algorithm used the following steps to determine switches and add-ons:

Step 1) For each individual and separately for each ASM, the algorithm assesses the start and the end of the supply of each redeemed prescription during the follow-up period, which period, for a convention, was defined as a medication event. The start of each medication event was defined as the date on which an individual redeemed the medication. The end of a medication event was calculated as the start of the medication event + the duration of the supply computed by the Sessa Empirical Estimator (SEE) (Meaidi et al., 2021; Pazzagli et al., 2022a; Pazzagli et al., 2022b).

Step 2) The algorithm calculates the periods of overlap of medication events of different ASMs during the follow-up period of each individual included in the study population.

Step 3) For all the potential combinations of ASMs with overlapping medication events during the follow-up period, the algorithm calculated the proportion of days of co-exposure from the total amount of potential co-exposure time (N). For all the potential combinations of ASMs co-redeemed during the follow-up period, the total amount of co-exposure time was calculated as the number of days from the first day of overlapping of medication events of different ASMs (i.e., different anatomical therapeutic chemical classification codes, *ATC* codes) to the last day in treatment with ASMs or the end of the observational window whichever comes first.

Step 4) For all the potential combinations of ASMs co-redeemed during the follow-up period, the algorithm calculated the time to the first co-exposure (J) of different ASMs as the number of days from the start of the follow-up period to the first day of co-exposure to the combination of interest.

Step 5) The algorithm used three different approaches for defining switching or add-on, and the approach with the best performance was used to determine if periods of co-exposure to different ASMs were switch and/or add-on.

Approach 1): We arbitrarily defined add-on to occur when the proportion of days of co-exposure (N) was ≥35%, while switching was defined as the proportion of days of co-exposure (N) ≤ 10%. Add-ons that are used for a short period are sensitive to misclassification with this approach. However, the issue has been solved by using pharmacological reasoning in approach 2 and then letting the algorithm learn from such reasoning in approach 3.

Based on approach 1, we classified add-on or switch to occur early during the observational window if it occurred between the start of the observational window and the first quartile of the total observational window (i.e., start ≤J≤ quartile 1). We defined an add-on or switch to occur late during the observational window if it occurred after the third quartile of the total observational window. We defined add-on or switch to occur in the middle of the observational window if it occurred between the first quartile of the total observational window and the third quartile of the total observational window.

**FIGURE 1:**
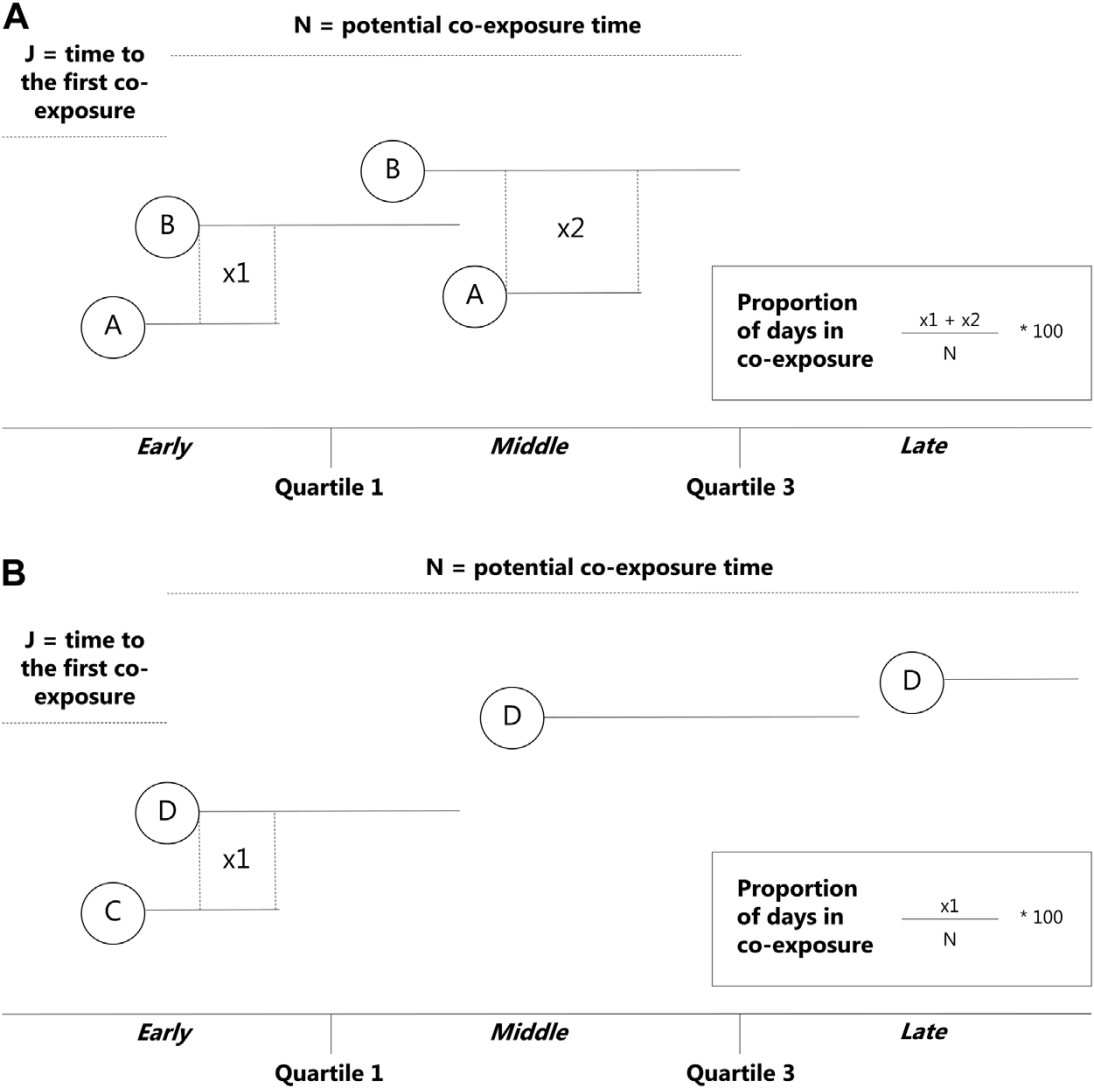
Overview of the newly developed algorithm—add-ons. N = potential co-exposure time from the beginning of the redeemed prescription of the second medication to the end of the follow-up period, J = time to the first co-exposure from the beginning of the observational window, used to determine which stage the co-exposure occurred in. **(A)** = drug 1, **(B)** = drug 2.

In this study, the early stage refers to those switches or add-ons that occurred between 0 and 182.5 days of the observational window. The middle stage refers to those switches or add-ons that occurred between 183 and 547.5 days of the observational window, and the late stage relates to those switches or add-ons that occurred ≥548 days of the observational window (Figures 1A,B).

Approach 2): In approach 2, we overcame the problem of misclassification of the add-on which was used for a short period by letting the algorithm classify as add-on co-exposure to medication events that notoriously follow these patterns (e.g., such as clonazepam and phenytoin). Clonazepam is used as a first-line treatment for myoclonic seizures and is used for a short period. Phenytoin is indicated as a co-drug in the treatment of convulsive status epilepticus; however, due to the potential for adverse drug reactions, phenytoin is not recommended to be used in combination with other ASMs for a long period (Iivanainen, 1998).

Approach 3): In approach 3, we used six different machine-learning models to classify individuals performing switching or add-on. In particular, we used the following: 1) linear regression, 2) naïve Bayes, 3) support vector machine, 4) neural network, 5) classification and regression tree, and 6) random forest. These methods have been described in detail in the context of pharmacoepidemiological research elsewhere (Sessa et al., 2020).

In all six models, we used 8-fold cross-validation assuring that all anti-seizure combinations were represented in the 8 folds. The models developed on the training set (75%) were subsequently used on the test set (25%) to assess the classification overall accuracy.

The following variables were used to train the models in the training set:

1) time to the first co-exposure from the beginning of the observational window,
2) the time from the first co-exposure to the end of the follow-up period,
3) the sequence of the switch/add-on, meaning the sequence of consecutive redeemed prescriptions with different ATC codes on a temporal scale.
4) the number of days from the date when individuals redeemed their first ASMs following epilepsy diagnosis until the end of the follow-up period, death, or emigration.
5) the year on which an individual redeemed the first ASM.
6) the duration of medication events computed by the SEE.
7) ICD_10_ code of the diagnosis of their first epilepsy diagnosis.
8) the period between the end of hospitalization for epilepsy and the first redeemed prescription of the ASM.
9) the duration of the hospitalization for epilepsy.

### 2.6 Data analysis

To identify if the algorithm correctly classified individuals as performing switching or add-on, we performed a manual inspection of the redeemed prescription pattern during the follow-up period. Plots were prepared by looking at medication event patterns for each individual included in the study population. Co-exposure periods to two ASMs were classified as a switch, add-on, or unknown using redeemed prescription pattern templates provided in Supplementary Figures S1–5.

We considered the manual revision the “real” truth from which we were able to compare the performance of the algorithm in classifying individuals as performing switching or add-on. The manual revision was revised by two pharmacoepidemiologists and the discrepancy was resolved by consensus after a discussion. Finally, we compared the classification performed by the algorithm with those obtained with the manual revision, and we built up a confusion matrix as described in Supplementary Tables S1,2.

From the confusion matrix, we computed the median overall classification accuracy and the 95% confidence intervals. Additional performance metrics (i.e., sensitivity and specificity) for each approach have been computed and shown in the supplementary material. For approach 3, we plotted the receiver operating characteristic (ROC) curve and computed the area under curve (AUC) for the model with the highest sensitivity and specificity.

For descriptive purposes, we plotted the density plot of the frequency of add-ons, switches, and unknowns performed during the observational window, overall, and stratified by the stage of the treatment. In addition, baseline characteristics of the study population such as age at the index date, sex, and the first redeemed ASMs were presented as proportion and frequency for categorical variables, and the median and interquartile range (IQR) for age. A boxplot of the follow-up period of the study population is provided.

### 2.7 Ethical aspects

According to Danish legislation (Law 502, 23 May 2018, 10), no approval from an Ethics Committee is needed. According to the legislation, permission is not needed for research based on registration data in Denmark. The dataset utilized in this research is an irreversibly anonymized version of one produced in 2018, with consent from the Regional Capital Area Data Protection Agency, the University of Copenhagen, and Statistics Denmark (project number 707278).

## 3 Results

### 3.1 Study population

In total, 15870 individuals were included in the study population as they were aged 65 years or older, were hospitalized for epilepsy, and received their first ASM following the hospitalization for epilepsy. The study population was composed of 52.0% men and 48.0% females which had a median age of 72.9 years (IQR: 65.6–80.2 years). In Supplementary Table S3, we presented the ICD_10_ codes of the diagnosis of epilepsy. Unspecified epilepsy was the most frequently reported diagnosis in the study population (9431 out of 15870, 59.4%), followed by focal epilepsy with a complex attack or generalized tonic–clonic status epilepsy (2454 out of 15870, 15.5%), and focal epilepsy with only simple focal attacks (936 out of 15870, 5.9%). The median duration of the hospitalization for epilepsy was 5 days with an IQR of 1–21 days.

The medications shown in Supplementary Table S4 are the first ASMs redeemed by the study population following their first hospitalization for epilepsy. The majority of individuals redeemed valproic acid (34.6%), lamotrigine (21.8%), oxcarbazepine (18.0%), or levetiracetam (10.1%), all of which are recommended as first-line medications for individuals aged 65 years or older in clinical guidelines.

The study population redeemed their first prescription ASM within the first 8 days from the admission date for their first hospitalization of epilepsy (IQR: 3–36 days).

### 3.2 Medication events with overlapping co-exposure time

During the first 730 days of the follow-up period, 988 patients from the study population out of 15870 underwent a total of 1485 medication events with co-exposure time to two or more ASMs. The median number of medication events with co-exposure to ≥2 ASMs per patient was one with an IQR of 1–2 and a maximum number of nine. Phenytoin/oxcarbazepine (162 out of 1485, 10.9%) was the most frequently observed combination, followed by phenytoin/lamotrigine (147 out of 1485, 9.9%), phenytoin/valproic acid (133 out of 1485, 9.0%), clonazepam/lamotrigine (128 out of 1485 8.6%), and clonazepam/oxcarbazepine (124 out of 1485, 8.4%) (Table 1).

**TABLE 1:** Number of individuals with co-exposure to two or more anti-seizure medications.

Medication	Number of individual
Phenytoin/oxcarbazepine	162
Phenytoin/lamotrigine	147
Phenytoin/valproic acid	133
Clonazepam/lamotrigine	128
Clonazepam/oxcarbazepine	124
Phenytoin/levetiracetam	118
Clonazepam/valproic acid	117
Clonazepam/levetiracetam	108
Carbamazepine/valproic acid	78
Carbamazepine/oxcarbazepine	76
Carbamazepine/lamotrigine	59
Carbamazepine/phenobarbital	41
Phenytoin/clonazepam	37
Phenytoin/phenobarbital	36
Clonazepam/phenobarbital	20
Clonazepam/carbamazepine	18
Phenytoin/carbamazepine	14
Carbamazepine/levetiracetam	14
Clonazepam/gabapentin	14
Clonazepam/topiramate	12
Phenytoin/topiramate	11
Carbamazepine/gabapentin	10

^a^ The same individual can appear in multiple groups as they can be co-exposed to two or more different anti-seizure medications during the follow-up period.

### 3.3 Manual revision

After the manual revision, we found 1065 add-ons among the 1485 medication events that occurred during the observation window (885 in the early stage, 140 in the middle stage, and 40 in the late stage). The most frequently used add-ons in the early stage were phenytoin/lamotrigine (108 out of 885, 12.2%), phenytoin/oxcarbazepine (106 out of 885, 12.0%) followed by phenytoin/levetiracetam (87 out of 885, 9.8%), clonazepam/lamotrigine (79 out of 885, 8.9%), and phenytoin/valproic acid (72 out of 885, 8.1%). Levetiracetam/clonazepam (20 out of 140, 14.3%) was the most frequently used add-on in the middle stage followed by valproic acid/clonazepam (16 out of 140, 11.4%), valproic acid/phenytoin (12 out of 140, 8.6%), lamotrigine/clonazepam (9 out of 140, 6.4%), and oxcarbazepine/clonazepam (8 out of 140, 5.7%). The most used add-ons in the late-stage were valproic acid/clonazepam (7 out of 40, 17.5%). The majority of add-ons (885 out of 1,065, 83.1 percent) occurred in the first 6 months (early stage) (Table 2; Supplementary Figure S6).

**TABLE 2:** Manual revision of medication events to identify add-ons occurred in the early, middle, and late phases of the follow-up period.

Medication (first drug/add-on)	Early stage (N = 885)	Middle stage (N = 140)	Late stage (N = 40)
Phenytoin/lamotrigine	108	*	*
Phenytoin/oxcarbazepine	106	*	*
Phenytoin/levetiracetam	87	*	*
Clonazepam/lamotrigine	79	*	*
Phenytoin/valproic acid	72	*	*
Clonazepam/oxcarbazepine	50	*	*
Clonazepam/levetiracetam	46	6	*
Clonazepam/valproic acid	38	*	*
Valproic acid/clonazepam	36	16	7
Phenytoin/clonazepam	30	*	*
Levetiracetam/clonazepam	28	20	*
Oxcarbazepine/clonazepam	26	8	*
Lamotrigine/clonazepam	18	9	*
Valproic acid/phenytoin	17	12	*
Phenytoin/phenobarbital	16	*	*
Lamotrigine/phenytoin	14	7	*
Oxcarbazepine/phenytoin	12	*	*
Carbamazepine/lamotrigine	11	*	*
Levetiracetam/phenytoin	10	6	*
Clonazepam/carbamazepine	8	*	*
Carbamazepine/phenobarbital	7	*	*
Phenobarbital/carbamazepine	7	*	*
Valproic acid/carbamazepine	6	*	*
**Additional information:** top 5 most frequently occurring add-ons in early, middle, and late stages, and they are as follows:			
Early add-on	Middle add-on	Late add-on	
Phenytoin/lamotrigine	Levertiracetam/clonazepam	Valproic acid/clonazepam	
Phenytoin/oxcarbazepine	Valproic acid/clonazepam	*	
Phenytoin/levertiracetam	Valproic acid/phenytoin	*	
Clonazepam/lamotrigine	Lamotrigine/clonazepam	*	
Phenytoin/valproic acid	Oxcarbazepine/clonazepam	*	

**N**. = number of add-ons. *we did not present the data when we had a frequency <6 individuals, accordingly to national law.

After the manual revision, we found 296 switches among the 1485 medication events that occurred during the observation window (246 in the early stage and 51 in the middle/late stage). Oxcarbazepine/carbamazepine was the most often used switch in the early stage (30 out of 249, 12.0%), followed by carbamazepine/valproic acid (26 out of 249, 10.4%), carbamazepine/lamotrigine (23 out of 249, 9.2%), carbamazepine/oxcarbazepine (20 out of 249, 8.0%), and oxcarbazepine/phenytoin (18 out of 249, 7.2%). Carbamazepine/valproic acid (8 out of 50, 16.0%) was the most commonly prescribed switch in the middle stage, followed by oxcarbazepine/carbamazepine (6 out of 50, 12.0%) and carbamazepine/oxcarbazepine (6 out of 50, 12.0%). The majority of the switch (245 out of 296, 82.8%) occurred in the first 6 months (early stage) (Table 3; Supplementary Figure S7). A boxplot of the follow-up period of the study population is provided in Supplementary Figure S8.

**TABLE 3:** Manual revision of switch medication events—phases: early, middle, and late.

Medication (first drug/switch)	Early stage (N = 245)	Middle and late stages (N = 51)
Oxcarbazepine/carbamazepine	30	6
Carbamazepine/valproic acid	26	8
Carbamazepine/lamotrigine	23	*
Carbamazepine/oxcarbazepine	20	6
Oxcarbazepine/phenytoin	18	*
Phenobarbital/carbamazepine	11	*
Phenytoin/oxcarbazepine	10	*
Oxcarbazepine/clonazepam	8	*
Valproic acid/carbamazepine	7	*
Carbamazepine/phenobarbital	6	*
Lamotrigine/carbamazepine	6	*
Phenytoin/valproic acid	6	*
Phenobarbital/phenytoin	6	*
Valproic acid/phenytoin	6	*
Valproic acid/clonazepam	6	*

**N**. = number of switches. *we did not present the data when we had a frequency <6 individuals, as, accordingly to national law, we cannot present these results.

After the manual revision, we found 124 medication events that were unclassifiable as switches or add-ons (91 in the early stage, 16 in the middle stage, and 17 in the late stage) (table 4).

**TABLE 4:** Manual revision of unknown medication events—phases: early, middle, and late.

Medication (change of unknown from the first mentioned to the second mentioned medication)	Early stage (N = 91)	Middle stage (N = 16)	Late stage (N = 17)
Oxcarbazepine/clonazepam	10	*	*
Valproic acid/carbamazepine	6	*	*
Phenytoin/valproic acid	6	*	*
**Additional information:** top 5 most frequently occurring unknowns in early, middle, and late stages, and they are as follows:			
Early unknown	Middle unknown	Late unknown	
Oxcarbazepine/clonazepam			
Valproic acid/clonazepam			
Phenytoin/valproic acid			

**N**. = number of unknowns. *we did not present the data when we had a frequency <6 individuals, accordingly to national law.

### 3.4 Algorithm performance

#### 3.4.1 Approach 1

The overall accuracy of the algorithm’s performance in classifying a switch was 66% (95% CI 64%–69%) (other performance metrics are provided in Supplementary Table S5) and for the add-on, it was 46% (95% CI 44%–49%) (other performance metrics are provided Supplementary Table S6). Approach 1 always classified correctly the direction of add-ons and switches, and the stage.

#### 3.4.2 Approach 2

The overall accuracy for the algorithm’s performance in classifying a switch was 88% (95% CI 0.86%–0.89%) (other performance metrics are provided in Supplementary Table S7) and for the add-on, it was 77% (95% CI 0.72%–0.80%) (other performance metrics are provided in Supplementary Table S8). Approach 2 always classified correctly the direction of add-ons and switches, and the stage.

#### 3.4.3 Approach 3

**FIGURE 2:**
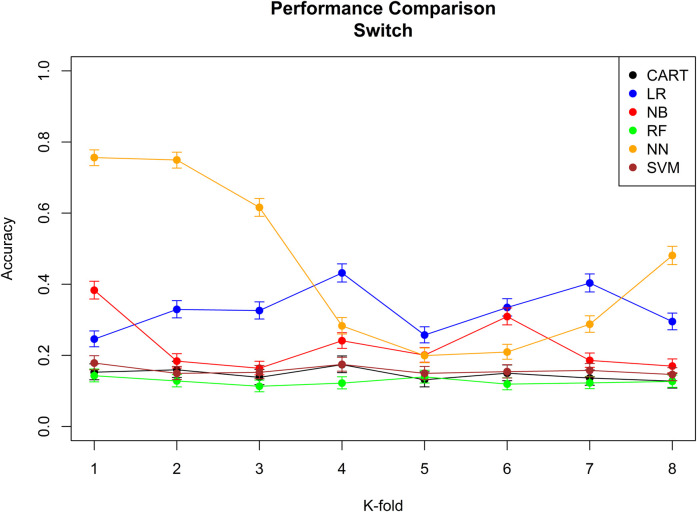
Performance comparison of all the six different machine learning/deep learning models used in approach 3 and the model’s accuracy of correctly classifying a switch. CART = classification and regression tree, LR = linear regression, NB = naïve Bayes, RF = random forest, NN = neural network, and SVM = support vector machine*.*

**FIGURE 3:**
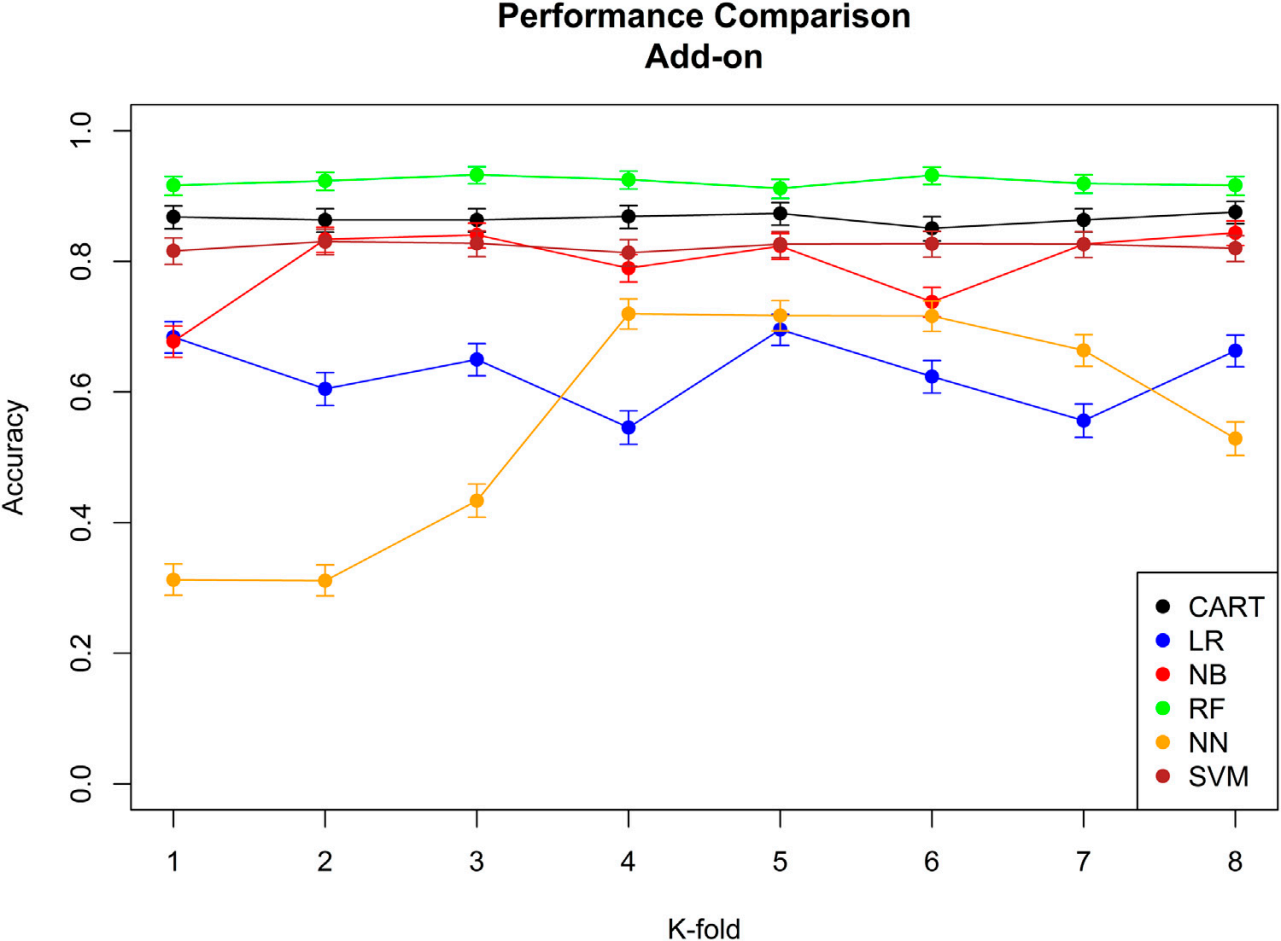
Performance comparison of all the six different machine learning/deep learning models used in approach 3 and the model’s accuracy of correctly classifying an add-on. CART = classification and regression tree, LR = linear regression, NB = naïve Bayes, RF = random forest, NN = neural network, and SVM = support vector machine*.*

The performance of the six machine learning/deep learning models is provided in Figures 2, 3, table 5, in which, we provided an overview of the 8 k-fold cross-validation results for the training and the test (named validation in the figures) sets.

**TABLE 5:** Summary of all the median accuracies of the six different machine learning/deep learning models used in approach 3.

Model	Switch (median overall accuracy)	Add-on (median overall accuracy)
Linear regression	0.33 (33%)	0.64 (64%)
Naïve Bayes	0.19 (19%)	0.82 (82%)
Support vector machine	0.15 (15%)	0.83 (83%)
Classification and regression tree	0.38 (38%)	0.86 (86%)
Neural network	0.38 (38%)	0.60 (60%)
Random forest	0.12 (12%)	0.92 (92%)

The model with the best performance for the add-on classification was the random forest with a median accuracy of 0.92 (92%), and the models with the best performance for switch classification were classification and regression tree and neural network, both with a median accuracy of 0.38 (38%). Approach 3 always correctly classified the direction of switches/add-ons and the stage. Median sensitivity and specificity for each machine-learning model, the ROC curve, and AUC for the model with the best sensitivity and specificity are provided in Supplementary Figure 9; Supplementary Tables 9,10.

Based on the overall accuracy performance of the three approaches, the best model for switches was provided by approach 2, and the best model for add-ons was the random forest method in approach 3. For both switches and add-ons, in 9 out of 10 medication events, the algorithm performed the correct classification.

## 4 Discussion

This study aimed at developing a new algorithm to identify switches and add-ons in secondary data sources with a focus on ASM users aged 65 years or older identified in Danish registers in the period between 1996 and 2018. Three different approaches of the algorithm were tested on 15,870 individuals hospitalized for epilepsy and on those who received their first ASM following the hospitalization for epilepsy.

The study population identified in Danish registers had similar demographic characteristics compared to other populations described in epidemiological studies conducted using data from other European countries and/or overseas countries (Sen et al., 2020). The majority of individuals enrolled in the study population had focal epilepsy, which is expected considering that the typical etiology of epilepsy in older individuals is stroke which notoriously causes focal epilepsy. Also, the duration of the hospitalization for epilepsy was in line with other epidemiological studies with a median of 5 days and an IQR of 1–21 days (Goldenberg, 2010).

The majority of individuals exposed to ASMs for the first time following a diagnosis of epilepsy redeemed valproic acid, lamotrigine, oxcarbazepine, or levetiracetam. This was expected as these medications are recommended as the first-line treatment of seizures in individuals aged 65 years or older by international guidelines (Sundhedsstyrelsen, 2014). The Danish guidelines recommend lamotrigine and levetiracetam as first-line medications for this subgroup. This result is in line with other epidemiological studies investigating the first received ASMs among epilepsy users emphasizing the comparability of the identified sample of ASM users in Danish registries with others from other European and overseas countries published in the scientific literature (Kwan and Brodie, 2001).

On average, the study population redeemed the first ASM within 3 days from the discharge. This is not surprising considering that in Denmark, health-care providers will not let an individual with epilepsy leave the hospital without available medication which will be provided in sufficient amounts to cover the period from the discharge until the patient redeems the medication from the pharmacy.

It was surprising to observe that during the first 730 days of the follow-up period, only 6.2% of patients from the study population (988 out of 15870) underwent to switches and/or add-ons. In previous studies, 27% of carbamazepine patients changed treatment due to the incidence of adverse events, while 13% of patients treated with valproic acid and 10% of lamotrigine users underwent a switch of treatment for therapeutic inefficacy (Kwan and Brodie, 2001). A plausible explanation for the low incidence of switches and add-ons could be the short follow-up period considered in this study. However, there could be an alternative reason. A recent study found a very high overall mortality among ASM users aged 65 years during the first year of treatment (Liang et al., 2021). This is an important finding as it is not possible to observe a switch/add-on if an individual dies during the follow-up period, and therefore, the high overall mortality may explain the aforementioned low incidence observed in this study (Liang et al., 2021).

Most of the switches (90.9%) and add-ons (70.9%) occurred in the early stage of the therapy (range: 0–182 days) which is not surprising. Previous studies have observed that only half of the newly diagnosed epilepsy patients will achieve a seizure-free status with the first ASM they use during the first 6 months of follow-up, which means that early during the treatment, there is a need for the health-care provider to prescribe another ASM and test what is effective and what is not effective in achieving a seizure-free status (Christopher Melinosky, 2021).

In the study population, on average, individuals performed one switch or add-on. However, there were also individuals who performed multiple switches and add-ons (up to 9). It was expected that those individuals having a high frequency of switches and add-ons had them in the late stage of the treatment as several switches and add-ons can only occur after multiple pharmacological attempts from health-care professionals, which, in turn, require time.

Regarding the algorithm performance, it is clear from the overall accuracy in approach 1 that this approach was not successful. One of the reasons for such a result is the extensive use of short-term add-on, which the algorithm wrongly classifies as switches. The problem of short-term add-on was solved by integrating pharmacological reasoning to approach 2. By doing so, it was possible to improve the overall accuracy of the algorithm from 0.66 (66%) to 0.88 (88%) for add-ons and from 0.46 (46%) to 0.77 (77%) for switches.

We believed that from the data, it was possible for machine learning/deep learning models to improve the classification performance of the algorithm as this model learned from the way the health-care providers prescribed the medication, the properties of these prescriptions, and the characteristics of the individual receiving the medication. In fact, it was not surprising to see that with a supervised learning approach, the algorithms’ overall accuracy improved for the add-on from 0.88 (88%) to 0.92 (92%). For switches, we observed no improvement by using a supervised learning approach. We believe that the main reason for such poor improvement for switches is the sparsity of data. In fact, only a few individuals performed switches, and therefore, machine learning/deep learning models did not have enough data to learn and therefore improve the classification accuracy.

It should be noted that add-ons have previously used machine learning to predict exposure to multiple distinct ASM as a proxy for drug-resistant epilepsy in administrative databases. However, our novel approach provides analytical advantages compared to the aforementioned as we were able to assess the period of co-exposure to multiple ASM. In addition, in our setting, the approach proposed was not feasible as counting only distinct ASM over a follow-up period does not provide any valuable information when the final goal of the study is assessing switch or add-on (Devinsky et al., 2016b; An et al., 2018).

### 4.1 Limitations

Medication events that did not overlap during the follow-up period were not assessed as they were considered stops of ongoing treatments and starts of new pharmacological treatments. In addition, switching to different strengths/formulations or different brand names of the same active ingredient was not considered in this algorithm. Combinations of ASMs with <6 individuals were excluded from the analysis as, due to national law, we could not present these results. A significant limitation of our data sources is that they lack reasoning on drug changes; thus, the ground truth of add-on or switching to second anti-seizure medication is unknown. Classification based on manual examination of drugs’ dispensing patterns may not be an accurate surrogate for the ground truth of the regimen type, especially when a drug is prescribed as add-on therapy but discontinued shortly after commencement due to adverse events. Moreover, 18 patients were diagnosed with “G402D—Severe myoclonic epilepsy in the childhood,” which we believe is a coding error.

## 5 Conclusion

This study provides a novel method to assess switches and add-ons in secondary data sources using population-wide health-care databases. The majority of switches and add-ons occurred early during the first 2 years of disease and according to clinical recommendations. The newly developed algorithm was able to classify correctly 9 out of 10 switches/add-ons. This is a very important achievement as population health-care databases usually lack granular information on drug changes. If the algorithm can accurately classify the regimen type (mono or combined therapy) or can be applied to subsequent regimens also in other therapeutic settings, it can greatly facilitate optimal use of large population health-care databases and better conduct studies on drug utilization or even help assess long-term treatment outcomes on a population basis.

## Data Availability

The data analyzed in this study are subject to the following licenses/restrictions: National laws. Requests to access these datasets should be directed to https://www.dst.dk/da/.
