# Supplementary material for: A machine-learning guided method for predicting add-on and switch in secondary data sources: A case study on anti-seizure medications in Danish registries
Source: Front Pharmacol. 2022 Nov 10;13:954393. doi: 10.3389/fphar.2022.954393 (PMC9685793; doi:10.3389/fphar.2022.954393)
Supplement: Supplementary file 1 [file Table1.docx]

**SUPPLEMENTARY MATERIAL**


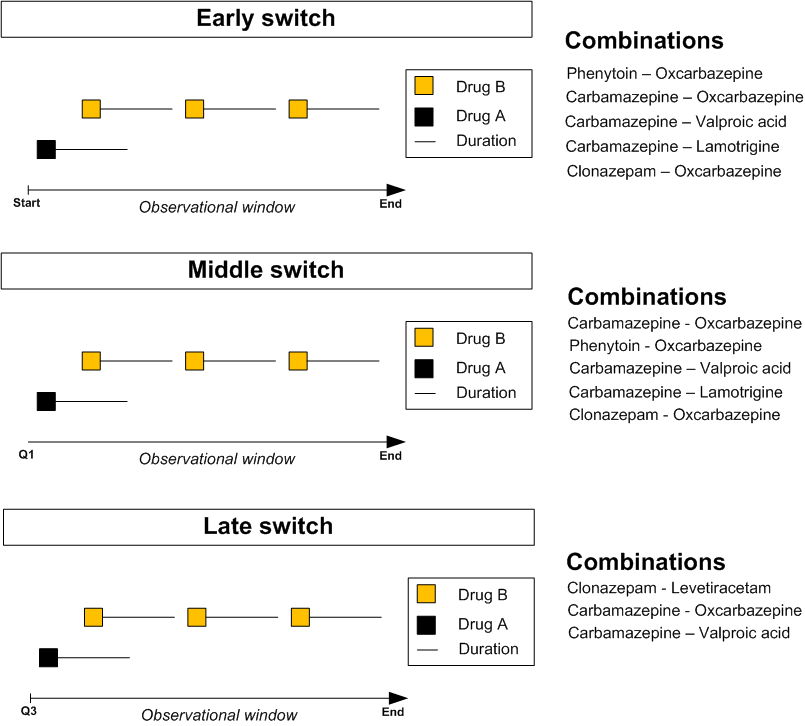


**Supplementary Figure 1.** A representative example of switching patterns from drug A to drug B in the early, middle, and late stages of the observational window. The pattern shows a hypothetical individual who initially redeemed the medication “Drug A” (black square) and switched to “Drug B” (yellow square). Combinations of the medications used in the early, middle, and late stages switches that follow this pattern are shown in the figure. Squares represent medication events and lines are their related duration of supply.


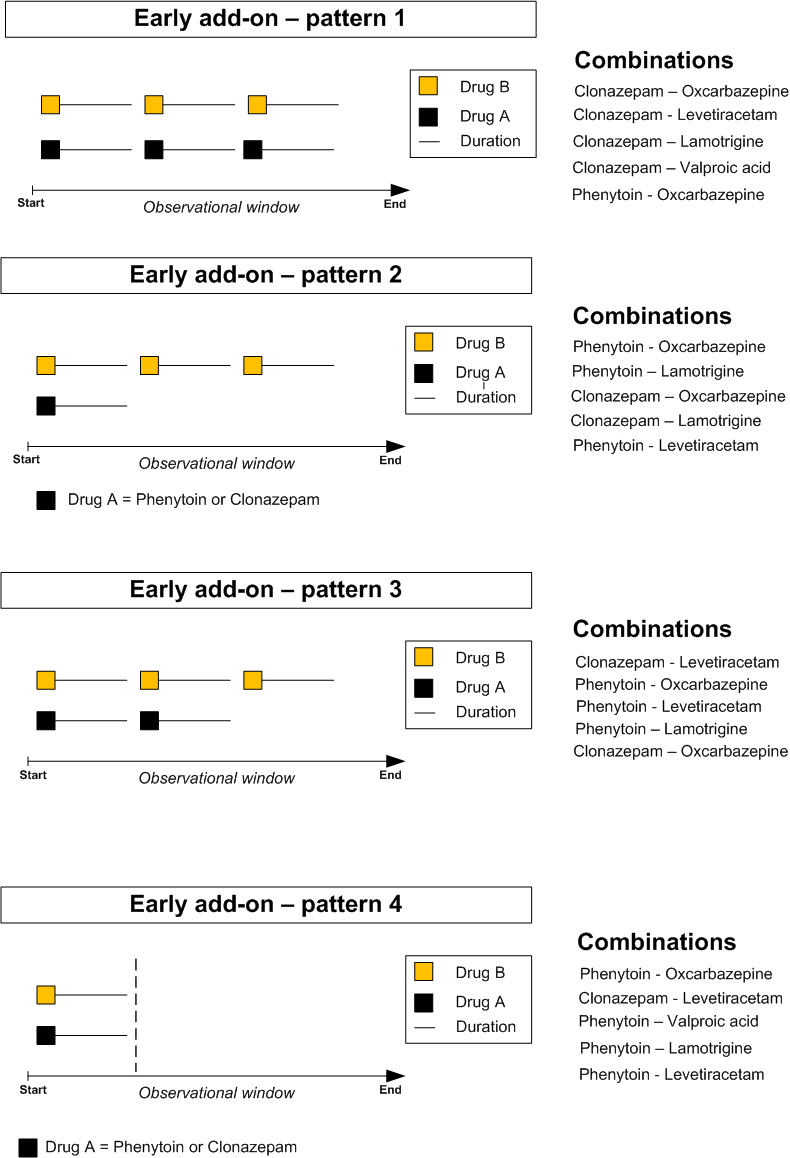


**Supplementary Figure 2.** A representative example of add-on patterns from drug A to drug B in the early stage of the observational window. The dotted vertical line in early add-on pattern 4 illustrates the end of the follow-up window. The pattern shows a hypothetical individual who initially redeemed the medication “Drug A” (black square) and received “Drug B” (yellow square) as an add-on. Combinations of the medications used in the early stage add-ons that follow this pattern are shown in the figure. Squares represent medication events and lines are their related duration of supply.


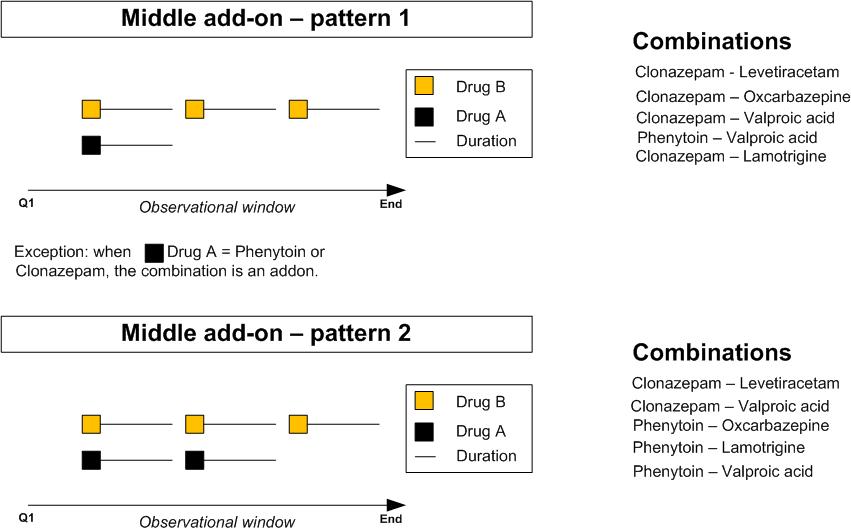


**Supplementary Figure 3.** A representative example of add-on patterns from drug A to drug B in the middle stage of the observational window. The pattern shows a hypothetical individual who initially redeemed the medication “Drug A” (black square) and received “Drug B” (yellow square) as an add-on. Combinations of the medications used in the middle stage add-ons that follow this pattern are shown in the figure. Squares represent medication events and lines are their related duration of supply.


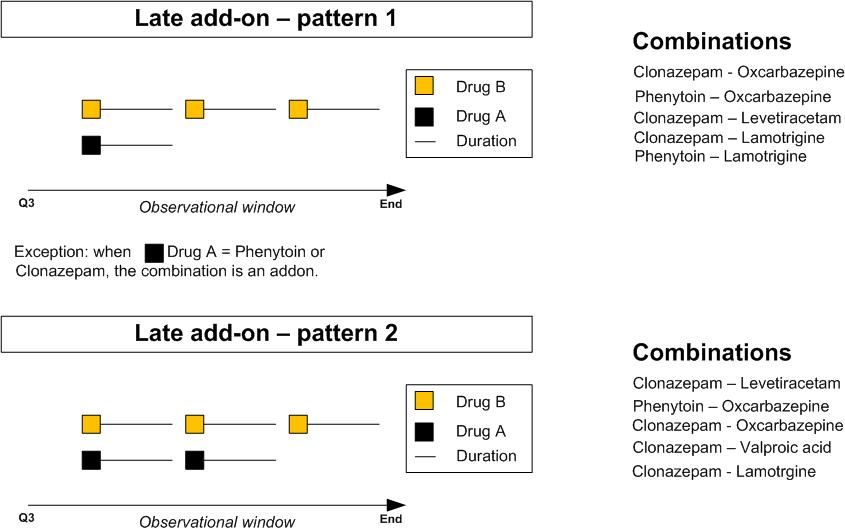


**Supplementary Figure 4.** A representative example of add-on patterns from drug A to drug B in the late stage of the observational window. The pattern shows a hypothetical individual who initially redeemed the medication “Drug A” (black square) and received “Drug B” (yellow square) as an add-on. Combinations of the medications used in the late stage add-ons that follow this pattern are shown in the figure. Squares represent medication events and lines are their related duration of supply.


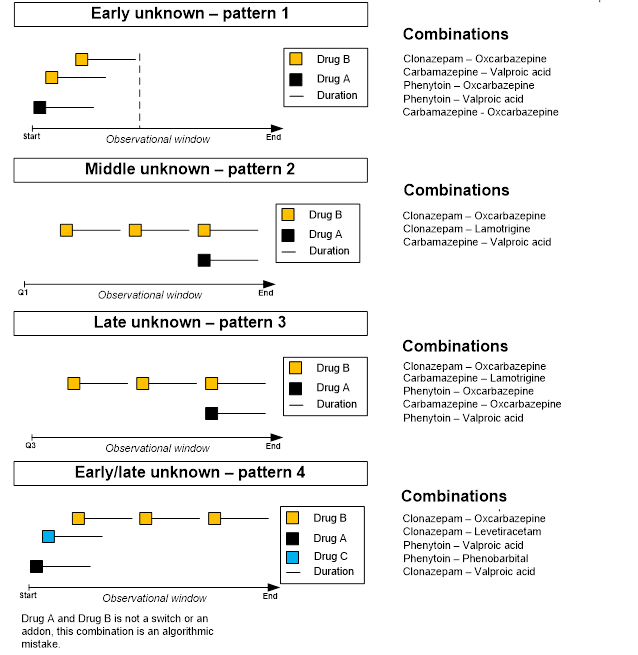


**Supplementary Figure 5.** A representative example of unknown patterns from drug A to drug B in the early, middle, and late stages of the observational window The pattern shows a hypothetical individual who initially redeemed the medication “Drug A” (black square) and received also “Drug B” (yellow square) and “Drug C” (blue square). Combinations of the medications used in the different stages of the observational window and that were classified as “unknown” that follow this pattern are shown in the figure. In patterns 1, 2, and 3, co-exposure was classified as unknown as there was not sufficient follow-up period to identify if the individual was performing a switch or rather an add-on. In pattern 4, co-exposure to “Drug C” was classified as unknown as the medication event can be either a short-term add-on or a switch.  Squares represent medication events and lines are their related duration of supply.


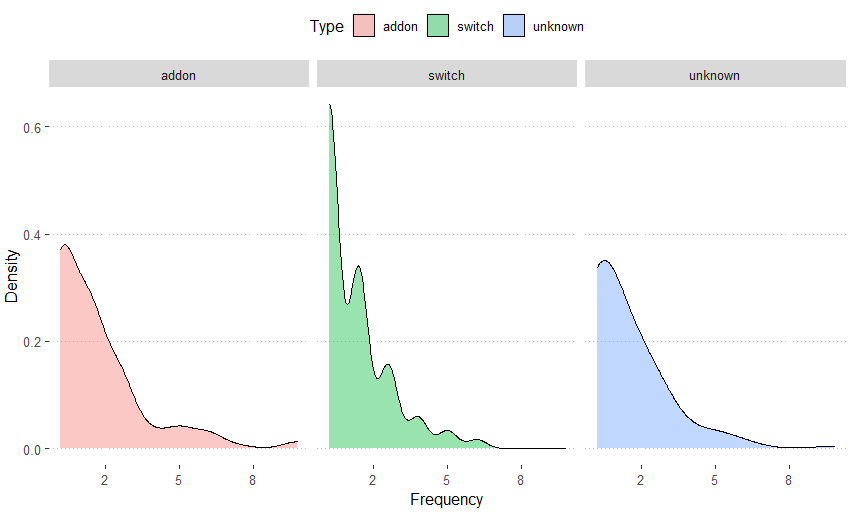


**Supplementary figure 6**. Illustrates the density of frequency of add-on, switch and unknowns where levels with >6 occurrences are presented.


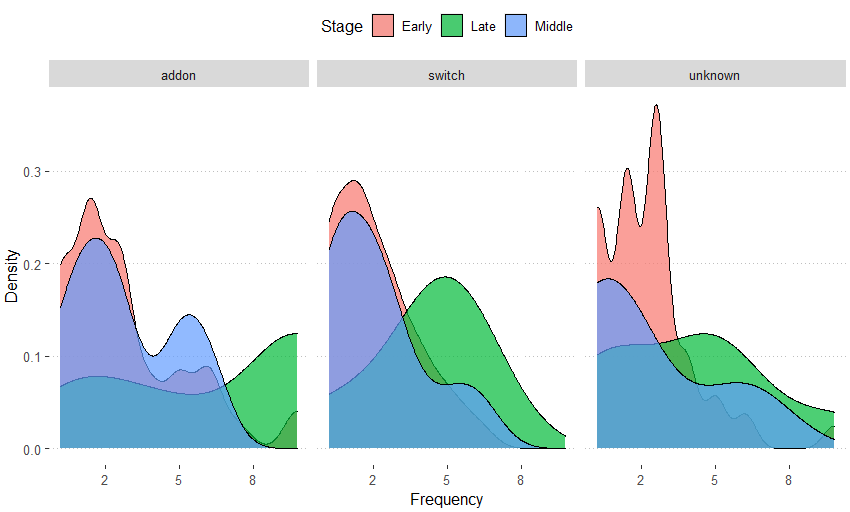


**Supplementary figure 7**. Represents the density of frequency of add-on, switch and unknowns in early, middle and late stages, only levels with >6 are presented in the figure.


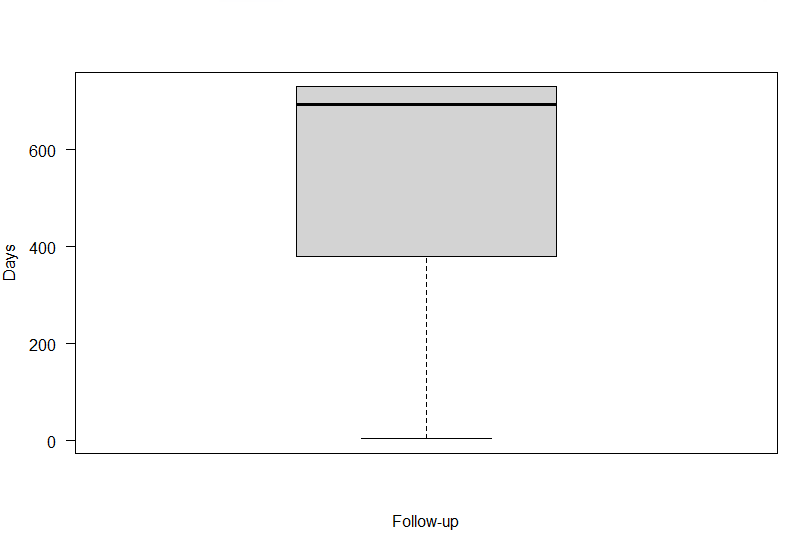


**Supplementary figure 8**. Boxplot of the follow-up period (days) of the study population.


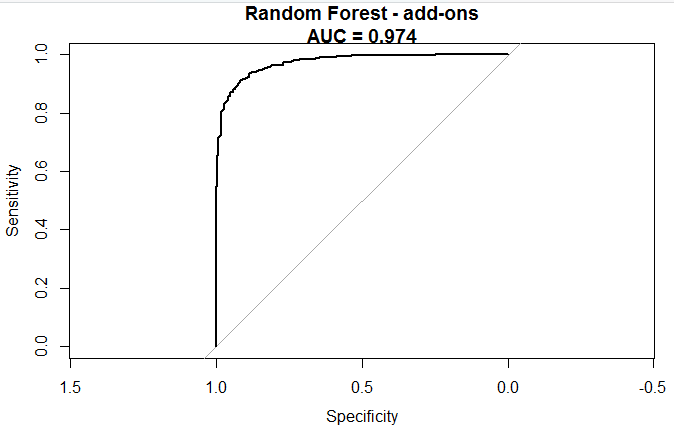


**Supplementary figure 9**. ROC curve and AUC for Random Forest when predicting add-ons.

**Supplementary Table 1**. A confusion matrix produced by counting the number of correct classifications performed by the new algorithm when compared to a gold standard method which in this study was considered the manual revision.

|  | | **Manually revised** | |
| --- | --- | --- | --- |
|  |  | *Switch* | *Others* |
| **Classified by the algorithm** | *Switch* | A | B |
|  | *Others* | C | D |

_A is the number of switch correctly classified by the new algorithm when compared with the manual revision performed by me and my supervisor._
_B is the number of switch wrongly classified by the new algorithm when compared with the manual revision performed by me and my supervisor.
C is the number of others wrongly classified by the new algorithm when compared with the manual revision performed by me and my supervisor.
D is the number of others correctly classified by the new algorithm when compared with the manual revision performed by me and my supervisor.
Others = an add-on or an unknown._

The formula for the overall accuracy in supplementary table 1 for switch used in Approach 2:
 $\frac{A+D}{Switch+Others}= \frac{A+D}{A+D+C+B}$

**Supplementary Table 2**. A confusion matrix produced by counting the number of correct classifications performed by the new algorithm when compared to a gold standard method which in this study was considered the manual revision.

|  | | **Manually revised** | |
| --- | --- | --- | --- |
|  |  | *Add-on* | *Others* |
| **Classified by the algorithm** | *Add-on* | A | B |
|  | *Others* | C | D |

_A is the number of add-on correctly classified by the new algorithm when compared with the manual revision performed by me and my supervisor._
_B is the number of add-on wrongly classified by the new algorithm when compared with the manual revision performed by me and my supervisor.
C is the number of others wrongly classified by the new algorithm when compared with the manual revision performed by me and my supervisor.
D is the number of others correctly classified by the new algorithm when compared with the manual revision performed by me and my supervisor.
Others = an add-on or an unknown._

The formula for the overall accuracy in supplementary table 2 for add-on used in Approach 2:
 $\frac{A+D}{Add-on+Others}= \frac{A+D}{A+D+C+B}$

**Supplementary Table 3**. ICD_10_ of the diagnosis of epilepsy.

| **ICD10 codes** | **N.** | **%** |
| --- | --- | --- |
| G409 – Unspecified epilepsy | 9431 | 59.5 |
| G402 – Focal epilepsy with complex attack or generalized tonic-clonic status epilepsy | 2454 | 15.6 |
| G401 – Focal epilepsy only with simple focal attacks | 936 | 5.9 |
| G406 – Unspecified Generalized tonic-clonic status epilepsy | 615 | 3.9 |
| G40 – Epilepsy | 436 | 2.8 |
| G408 - Other epilepsy and recurrent seizures | 420 | 2.6 |
| G403 – Generalized idiopathic epilepsy | 310 | 2.0 |
| G404 – Encephalopathy epilepsy | 280 | 1.8 |
| G400 – Idiopathic focal epilepsy | 245 | 1.6 |
| G419 – Unspecified status epilepsy | 194 | 1.2 |
| G410 – Generalized tonic-clonic status epilepsy | 128 | 0.8 |
| G405 – Other epilepsies | 123 | 0.8 |
| G418 – Another form of Status epilepsy | 48 | 0.3 |
| G412 – Non-convulsive complex partial status epilepsy | 45 | 0.3 |
| G407 – Unspecified absence type | 35 | 0.2 |
| Other diagnosis | 30 | 0.2 |
| G409A – Unspecified epilepsy with Generalized tonic-clonic status epilepsy | 23 | 0.1 |
| G400D – Autosomal dominant night frontal lobe epilepsy | 19 | 0.1 |
| G402D – Severe myoclonic epilepsy in the childhood | 18 | 0.1 |
| G411 – Non-convulsive status epilepsy of absence type | 14 | 0.1 |
| G402A – Frontal lobe epilepsy with complex focal seizure or generalized tonic-clonic status epilepsy | 13 | 0.1 |
| G401A - Frontal lobe epilepsy only with simple focal attacks | 12 | 0.1 |
| G405B – Reflex epilepsy | 9 | 0.05 |
| G401D – Temporal lope epilepsy only with simple focal attacks | 9 | 0.05 |
| G402B – Parietal lope epilepsy with complex focal epilepsy or generalized tonic-clonic status epilepsy | 8 | 0.05 |
| G403J – Sleep related epilepsy | 8 | 0.05 |
| G41 – Status epilepsy | 7 | 0.04 |

**N.** = number of individuals.

**Supplementary table 4.** First redeemed antiseizure medication following a hospitalization for epilepsy.

| **ATC code – active ingredient** | **N.** | **%** |
| --- | --- | --- |
| N03AG01- Valproic Acid | 5484 | 34.5 |
| N03AX09 - Lamotrigine | 3458 | 21.7 |
| N03AF02 - Oxcarbazepine | 2861 | 18.0 |
| N03AX14 – Levetiracetam | 1604 | 10.1 |
| N03AA02 - Phenobarbital | 821 | 5.2 |
| N03AF01 – Carbamazepine | 548 | 3.5 |
| N03AX12 – Gabapentin | 442 | 2.8 |
| N03AB02 - Phenytoin | 370 | 2.3 |
| N03AE01 – Clonazepam | 154 | 1.0 |
| N03AX16 - Pregabalin | 58 | 0.4 |
| N03AX11 – Topiramate | 44 | 0.3 |
| Other antiseizure medications | 17 | 0.1 |
| N03AA03 – Primidone | 9 | 0.1 |

**N.** = number of individuals. **ATC code =** Anatomical Therapeutic Chemical Classification System

**Supplementary Table 5.** Illustrates the amount of switch and others correctly/wrongly classified by approach 1 of the algorithm compared with the manual revision.

|  | | **Manually revised** | |
| --- | --- | --- | --- |
|  |  | Switch | Others |
| **Classified by the algorithm** | Switch | 236 | 440 |
|  | Others | 60 | 749 |

*Sensitivity=*0.7973; *Specificity=* 0.6299.

**Supplementary table 6.** Represents the amount of add-on and others correctly/wrongly classified by the algorithm compared with the manual revised in approach 1.

|  | | **Manually revised** | |
| --- | --- | --- | --- |
|  |  | Add-on | Others |
| **Classified by the algorithm** | Add-on | 306 | 41 |
|  | Others | 759 | 379 |

*Sensitivity=* 0.2873; *Specificity=* 0.9024.

**Supplementary table 7.** Illustrates the amount of switches and others correctly/wrongly classified by the algorithm compared with the manual revision in approach 2.

|  | | **Manually revised** | |
| --- | --- | --- | --- |
|  |  | Switch | Others |
| **Classified by the algorithm** | Switch | 236 | 123 |
|  | Others | 60 | 1066 |

*Sensitivity=*0.7973; *Specificity=*0.8966.

**Supplementary table 8.** Represents the amount of add-ons and others correctly/wrongly classified by the algorithm compared with the manual revised in approach 2.

|  | | **Manually revised** | |
| --- | --- | --- | --- |
|  |  | Add-on | Others |
| **Classified by the algorithm** | Add-on | 772 | 41 |
|  | Others | 293 | 379 |

*Sensitivity=* 0.7249; *Specificity=* 0.9024.

**Supplementary table 9.** Additional performance metrics for approach 3 – switch.

| **Model** | **Sensitivity** | **Specificity** |
| --- | --- | --- |
| CART | 0.203 | 0.120 |
| LR | 0.774 | 0.176 |
| NB | 0.274 | 0.144 |
| RF | 0.149 | 0.121 |
| NN | 0.267 | 0.534 |
| SVM | 0.392 | 0.085 |

**Supplementary table 10.** Additional performance metrics for approach 3 – add-on.

| **Model** | **Sensitivity** | **Specificity** |
| --- | --- | --- |
| CART | 0.932 | 0.731 |
| LR | 0.833 | 0.233 |
| NB | 0.907 | 0.683 |
| RF | 0.953 | 0.824 |
| NN | 0.468 | 0.681 |
| SVM | 0.934 | 0.517 |
